# Moving Past Anti-VEGF: Novel Therapies for Treating Diabetic Retinopathy

**DOI:** 10.3390/ijms17091498

**Published:** 2016-09-07

**Authors:** Mark T. Bolinger, David A. Antonetti

**Affiliations:** Departments of Ophthalmology and Visual Sciences, Kellogg Eye Center, and Molecular and Integrative Physiology, University of Michigan, Ann Arbor, MI 48105, USA; bolimark@umich.edu

**Keywords:** diabetic retinopathy, diabetic macular edema, cytokine, VEGF, TNF-α, blood retinal barrier, corticosteroid, renin-angiotensin system, kinin-kallikrein system, angiopoietin, vitreomacular adhesion

## Abstract

Diabetic retinopathy is the leading cause of blindness in working age adults, and is projected to be a significant future health concern due to the rising incidence of diabetes. The recent advent of anti-vascular endothelial growth factor (VEGF) antibodies has revolutionized the treatment of diabetic retinopathy but a significant subset of patients fail to respond to treatment. Accumulating evidence indicates that inflammatory cytokines and chemokines other than VEGF may contribute to the disease process. The current review examines the presence of non-VEGF cytokines in the eyes of patients with diabetic retinopathy and highlights mechanistic pathways in relevant animal models. Finally, novel drug targets including components of the kinin–kallikrein system and emerging treatments such as anti-HPTP (human protein tyrosine phosphatase) β antibodies are discussed. Recognition of non-VEGF contributions to disease pathogenesis may lead to novel therapeutics to enhance existing treatments for patients who do not respond to anti-VEGF therapies.

## 1. Introduction

Diabetic retinopathy (DR) is a serious threat to vision that is poised to become even more problematic with the increasing worldwide incidence of diabetes. Approximately 60% of individuals with type 2 diabetes and nearly all individuals with type 1 diabetes show at least some signs of DR [1,2]. In the USA, DR is the leading cause of blindness amongst working age adults (ages 20–74) [1] with an estimated treatment cost of $500 million annually in the United States alone [3,4]. In 2014, there were an estimated 422 million individuals with diabetes, up from 108 million in 1980 [5]. The current worldwide estimate of diabetic patients with any form of DR is 92.6 million, including 17.2 million with the more serious proliferative form of DR, 20.6 million with diabetic macular edema (DME) and a total of 28.4 million people with DR that is considered to be a threat to vision [6]. In the US, it is projected that by the year 2050 up to one third of the total adult population could have diabetes, leading to a vast increase in cases of DR [7]. The treatment costs, loss of productivity, decreased quality of life, and dramatically increased incidence emphasize the need for effective DR therapies.

## 2. Retinal Anatomy and the Blood Retinal Barrier

The human retina is a laminar tissue composed of five basic layers: three layers of neural somas separated by two neural synaptic plexuses [8] that may be considered as the inner and outer retina based on functional and morphological differences. Light travels through the largely transparent retina to the outer retina consisting of rod and cone photoreceptor cells that convert and transmit light information as neurochemical signals via synaptic connections in the outer plexiform layer to the neurons of the inner nuclear layer. Posterior to the outer nuclear layer is the outer limiting membrane, photoreceptor inner and outer segments, retinal pigmented epithelium (RPE), Bruch’s membrane, and the choroidal vasculature necessary to support the highly metabolically active rods and cones. The inner nuclear layer is composed of various cell types including bipolar, amacrine, and horizontal cells, which receive the transmitted signal from the rods and cones and relay this information through synaptic connections in the inner plexiform layer to the inner most ganglion cell layer, composed of displaced amacrine cells and ganglion cells. Finally, the axons of the ganglion cells create the optic nerve to the brain and traverse the lateral geniculate nucleus in the thalamus, ultimately leading to the occipital lobe where the signal is interpreted as vision [9].

### 2.1. The Blood Retinal Barrier

Mammals are the only animals with a vascularized retina. In primates and rodents, the inner retina receives vascular support from blood vessels that branch from the central retinal artery, ultimately traversing across the inner retina forming capillary plexuses in the ganglion cell layer and on either side of the inner nuclear layer [10]. Blood exits the retina from the central retinal vein that lies next to the central retinal artery at the optic disk. Similar to the brain and other neural tissues, the retina requires a barrier to blood borne material due to the requirement for a highly regulated environment for proper neuronal function and cellular interaction. Further, the retina is immune-privileged or at a minimum, restricted [11]. The blood-retinal barrier (BRB) describes the unique make-up of the retinal vasculature and RPE cells that tightly control the flux of fluid and blood borne material into and out of the retina and confer immunoprivilege. The inner BRB component is composed of a well-developed junctional complex between adjacent endothelial cells of the retinal vasculature that restrict paracellular flux and that have limited vesicle and fenestrae formation reducing transcellular permeability [12]. The cells also express efflux transporters of the multi-drug resistance family [13]. Like the brain, formation of these specialized endothelial cells requires interaction with glia and pericytes and recent studies have identified the contribution of Norrin signaling from glia as required for development and maintenance of the BRB [14]. The glia and pericytes provide endothelial support and protection, and their loss results in increased endothelial permeability [15]. Together, this complex is known as the neurovascular unit, and accumulating evidence suggests that disruptions of normal pericyte or glial interaction with the endothelium leads to loss of barrier properties (reviewed in [12,16,17,18]).

The retina is the most metabolically active bodily tissue and the rod and cone containing outer nuclear layer is the most metabolically active layer of the retina, accounting for at least half of its total metabolic activity [19,20]. However, unlike the inner retina, photoreceptors reside within the avascular environment of the outer retina, and metabolic activity is supported by diffusion and transport of blood borne material across the retinal pigmented epithelium (RPE) from the nearby choroidal vasculature. RPE cells have well developed junctional complexes between adjacent cells that restrict paracellular flux of potentially harmful substances, and maintain the sensitive retinal environment. Together the RPE and the specialized vasculature of the inner retina comprise the BRB.

### 2.2. Tight Junctions

Tight junctions (TJs) are specialized cell-cell contacts of the inner retinal vascular cells and RPE cells of the outer BRB and are part of the junctional complex that also includes the adherens junction. Over 40 TJ proteins have been identified to date, some of which are transmembrane and facilitate restriction of paracellular flux while others are cytosolic and serve as organizers of transmembrane proteins, connecting them to the cytoskeleton [21]. The result is a continuous junction that circumscribes the most apical portion of the lateral membrane in the RPE and connects the endothelial cells allowing formation of a protected lumen in the vascular endothelium. The junctional complex connects the cytoskeleton across the membrane to the TJ on adjacent cells [22]. Permeability is inversely correlated with content of the TJ protein, occludin, across many tissues [23,24], and gene deletion experiments have identified required TJ proteins necessary for barrier properties in different tissues [25]. The phenotypes of TJ protein gene deletion include embryonic lethality for ZO-1 [26], ZO-2 [27], and claudin-1 [28], aberrant permeability regulation (claudin 2 [29] and claudin 10 [30]), increased permeability (claudin 5 [31] and claudin 18 [32]), or various blood–barrier related pathologies (occludin [33,34]). Further, changes in occludin phosphorylation and ubiquitination are required for VEGF induced permeability in cell culture [35]. Genetic studies have clearly identified a role for post-translational modifications of the adhesion junction transmembrane protein, VE-cadherin, in permeability and inflammation in the blood-brain barrier [36]. Future studies using genetic analysis will be necessary to demonstrate a causative role for the junctional proteins in control of retinal disease pathology.

## 3. Diabetes-Associated Ophthalmic Pathologies

### 3.1. Diabetic Retinopathy

DR is classified as either non-proliferative or proliferative based on disease progression. Non-proliferative DR (NPDR) is characterized by increased permeability of retinal vessels, microaneurysms, exudate deposits, basement membrane thickening, and microhemorrhages [12,37]. Proliferative DR, or PDR, is more serious and presents with the same clinical symptoms as NPDR, but with the addition of pathologic angiogenesis. These new vessels are fragile and prone to leak fluid and blood borne material into the eye. Blood within the vitreous can obstruct the path of light to the retina, resulting in specks, or “floaters” within the visual field [38]. Additionally, neovascularization from the retina can penetrate the vitreous causing vitreoretinal traction and resulting in retinal detachment and blindness if not surgically repaired [37].

### 3.2. Diabetic Macular Edema

Diabetic macular edema (DME) can be a clinical feature of either the non-proliferative or proliferative form of DR, but is more common in the proliferative form [39], and poses a serious threat to vision [40]. After 10 years of disease, DME has incidence rates of 11% and 14% in type I and type 2 diabetes, respectively [41,42]. DME is the clinical correlate most closely associated with vision loss with retinal center point thickness measured by optical coherence tomography, and fluorescein leakage in retinal blood vessels, combined with age, accounting for 33% of the variation in visual acuity [43]. Clearly there is a need for additional biomarkers that better predict retinal dysfunction in DR. The macula is a region within the central retina of the primate eye that is characterized by extremely high cone density and lateral relocation of inner retinal bipolar and ganglion cells with no vascularization such that the path of light to photoreceptors is minimally obstructed. As a result of these characteristics, visual acuity in lighted conditions is higher in the macula than any other ocular region. DME is characterized by accumulation of fluid and blood borne material leading to subsequent thickening of the tissue and resulting in deleterious changes to macular optical properties and decreased visual acuity. Additionally, studies have demonstrated improved visual acuity following reduction in DME [44]. However, alterations in retinal structure are likely not the only cause of visual loss after DME, and changes to the proper ion environment necessary for neuronal transmission, neurotransmitter excitotoxicity, and inflammation have all been suggested as potential mechanisms of loss of proper retinal function associated with loss of the BRB. How loss of BRB goes beyond changing optical properties and leads to loss of proper neural function and potential neural cell death is an important gap in our current knowledge that demands greater attention. Nevertheless, DME remains the clinical correlate most closely associated with visual loss in both PDR and NDPDR [45,46,47].

Historically, DR and DME have been treated by improved glycemic control in the form of insulin sensitizers, vigilant exogenous insulin administration, or improved diet and exercise. Retinal photocoagulation, which was first shown to be therapeutically beneficial in 1954, uses a laser to cauterize or destroy pathological vessels [48,49]. It can either be applied evenly across the non-macular retina (panretinal photocoagulation), or at specific vessels (focal photocoagulation). Following its introduction, subsequent clinical trials confirmed its effectiveness leading to extensive use over many decades [49,50]. However, retinal photocoagulation generally only prevents further vision loss rather than restoring vision, and leads to side effects including reduced night and peripheral vision [51].

More recently, control of the underlying disease though intensive glycemic control has been stressed to reduce the incidence and severity of diabetic complications, including DR. The Action to Control Cardiovascular Risk in Diabetes (ACCORD) trial set forth a protocol which lowered the targeted glycated hemoglobin (HbA1c) in patients with type 2 diabetes to below 6.0% from a standard range of 7.0%–7.9% [52]. This was found to significantly reduce DR progression in patients under intensive compared to standard glycemic control [53]. The data concur with previous trials including the United Kingdom Prospective Diabetes Study (UKPDS) which demonstrated a decrease in DR progression in patients with type 2 diabetes [54], and the Diabetes Control and Complications Trial (DCCT), which reported a 76% decrease in the risk of developing DR, and 54% reduction in disease progression in patients with type 1 diabetes [55]. Additionally, the DCCT follow up, known as the Epidemiology of Diabetes Interventions and Complications (EDIC) study, found a decreased incidence of PDR and DME in the former DCCT intensive glycemic control group four years after the conclusion of DCCT, even though the observed differences in glycosylated hemoglobin had largely disappeared [56]. Together, these trials confirm the clinical efficacy of intensive glycemic control in preventing the incidence and progression of DR. It is important to note that these studies also highlight the need to prevent hypoglycemia as the intensive control group of the ACCORD trial revealed a 22% higher rate of all deaths than the standard group which led to termination of intensive treatment and transfer of all patients to the standard treatment [52].

## 4. Therapeutic Intervention

A number of potential therapies for diabetic retinopathy have been tested in clinical trials. The success of therapies targeting VEGF has spurred innovation and development of novel approaches to treat this blinding disease. Table 1 provides information on successful and ongoing clinical trials for therapies targeting diabetic retinopathy, and these therapies are discussed in detail below.

### 4.1. VEGF

One of the primary factors in the development of DR and DME is pathological release of VEGF. VEGF is a potent mitogen, identified in 1989 [57,58]. This cytokine is released by numerous cell types in response to hypoxic conditions, and was found to be increased in patients with PDR [59,60]. VEGF-mediated vascular growth relieves hypoxia and is a critical process in developmental vasculogenesis and angiogenesis, allowing for sufficient and uniform retinal vascular architecture [10]. In cases of DR/DME however, VEGF release results in pathological angiogenesis that is irregularly distributed and features poorly constructed vessels that are prone to leak, leading to fluid build-up within the retina. Increased vascular leakage is a serious risk factor for DME, and has been found to be a better predictor of progression to clinically significant DME than retinal vessel diameter or retinal thickness [46]. VEGF negatively affects TJ architecture within the BRB, and leads to endocytosis of key TJ proteins and subsequent increases in permeability [35,61].

Beginning in late 2004, a series of anti-VEGF treatments were granted FDA approval to treat neovascularization associated with wet age related macular degeneration and/or DR based on successful large, multicenter clinical trials (for VEGF reviews, see [62,63,64]). These include pegaptanib (2004), ranibizumab (2006, for neovascular age-related macular degeneration and 2015, for DR and DME), and aflibercept (2011). The landmark phase III RISE/RIDE trials reported a significant increase in visual acuity in ~45% of patients with DR/DME in response to anti-VEGF treatment [65]. Further, over a two-year window ranibizumab was recently shown to be noninferior to pan-retinal photocoagulation for patients with PDR, the standard of care for decades [66]. Additionally, the full-length anti-VEGF antibody bevacizumab was approved to treat colon cancer in 2004, but is widely used off-label for treatment of DR and DME due to its lower cost and similar efficacy to ranibizumab [67]. The development of topical VEGF receptor small molecule inhibitor is in progress with an ongoing preliminary clinical trial involving PanOptica’s PAN-90806. Additionally, multiple clinical trials have been conducted or are currently in progress examining combined anti-VEGF and laser therapy approaches.

### 4.2. TNF-α

Despite the success of anti-VEGF treatment, these studies indicate that significant numbers of patients experiencing DME and/or pathological angiogenesis do not respond to treatment, consistent with the involvement of non-VEGF mediators of disease in DR and DME [68,69]. As evidence for the role of various cytokines and growth factors within the diabetic retina accumulates, DR is increasingly being recognized as an inflammatory disease with contributions by inflammatory cytokines such as tumor necrosis factor alpha (TNF-α) [70]. TNF-α is implicated in increased BRB permeability, and possibly in the development of DME. Specifically, TNF-α is up regulated by transcriptional nuclear factor kappa B (NF-κB), and may lead to intracellular adhesion molecule-1 (ICAM-1) mediated endothelial leukocyte adhesion and endothelial dysfunction [71,72]. Both plasma and vitreous TNF-α levels are elevated in patients with diabetes, and plasma TNF-α levels are correlated with DR severity [73,74]. Additionally, BRB permeability is increased up to five days post intravitreal TNF-α injection along with evidence of macrophage activation in a rat model [75]. In TNF-α treated BREC, mRNA and protein levels of the TJ proteins ZO-1 and claudin-5 are decreased, along with mis-localization of both proteins and a corresponding increase in permeability [76]. At higher doses, TNF-α may reduce endothelial cell viability. Retinal apoptosis was decreased in two diabetic models of TNF-α knockout mice, suggesting that apoptosis may be a mechanism of TNF-α induced permeability [77].

Despite the evidence linking TNF-α to various DR related pathologies including inflammation and increased permeability, the results of several small clinical trials with anti-TNF-α treatments have been mixed at best. The anti-TNF-α drug infliximab has had limited success when administered systemically [78], but neither infliximab nor a second anti-TNF-α, adalimumab, has proved successful in patients by intravitreal injection [79]. Additionally, adverse inflammatory effects have been reported, particularly to infliximab, greatly decreasing therapeutic utility [79,80]. Current usage of anti-TNF-α treatments is primarily for uveitis, and future application of these drugs to DR/DME will need to be supported by large, multicenter clinical trials [81].

### 4.3. Corticosteroids

One of the first effective medical treatments for DR and related pathologies dating back to at least 1950 was the use of corticosteroids including cortisone and later dexamethasone and prednisone to control intraocular inflammation [82]. Inflammation is one of the early hallmarks of DR/DME, and aqueous humor analysis of patients shows that numerous cytokines and factors are increased [83]. Corticosteroids are particularly effective in that they are active against a range of inflammatory factors contributing to DME [84]. Administration was initially systemic. However, serious adverse side effects of systemic administration including osteoporosis and glucose intolerance were soon identified [82,83,84,85,86]. Intraocular injection also carries unwanted side effects including an increase in intraocular pressure (IOP), a risk factor for glaucoma and cataract formation [87,88,89,90]. However, some patients resistant to anti-VEGF may respond to corticosteroids due to multiple inflammatory cytokines in patients with DME [91].

Recent strategies to avoid unwanted complications from corticosteroids include development of slow release implants that can be positioned very near target tissues and deliver lower doses of drug. The development of intravitreal steroid-releasing implants has allowed lower doses to be used, reducing many of the harmful side effects. Non-human primate studies with the dexamethasone implant, Ozurdex (Allergan) confirmed therapeutic retinal and vitreous levels at two months post implant, and detectable levels up to six months [92]. In multicenter clinical trials enrolling over 1000 patients, Ozurdex was shown to significantly improve visual acuity in a dose dependent manner [93]. It received FDA approval for treatment of DME in 2014, but due to an increase in the incidence of cataract formation, this approval was restricted to specific cases, including patients who are planning cataract surgery, or who have an artificial lens [93]. Importantly, Ozurdex was found to significantly improve visual acuity and central foveal thickness in eyes that had not responded to anti-VEGF (ranibuzumab or bevacizumab) [94,95].

The effectiveness of the synthetic corticosteroid, fluocinolone, has also been demonstrated, and a surgically placed fluocinolone implant, Retisert (Bausch & Lomb), was approved for the treatment of uveitis in 2005. Retisert improved visual acuity in subsequent multicenter off-label trials of patients with DME, but also exhibited an elevated incidence of cataracts and glaucoma compared with Ozurdex that has limited its use [96,97,98]. As of 2014, a fluocinolone implant previously available in Europe, Iluvien (Alimera Sciences), is FDA approved for treatment of DME. The fluocinolone acetonide in human aqueous (FAMOUS) trials enrolled 37 patients and determined that Iluvien delivered measurable levels of fluocinolone for at least three years, with the highest concentrations observed over the first three months [99]. This was combined with a significant improvement in visual acuity at three months with low dose, three and six months with high dose, and center point thickness at three, six, and twelve months for both doses [100]. While the improvement in visual acuity was increased at the higher dose, it was also associated with an increase in IOP [100]. FDA approval for treatment of DME was granted in 2014, but restricted to patients with a corticosteroid treatment history and no history of significant rise in IOP. In summary, injectable or surgically delivered intraocular corticosteroid implants are effective for treating DME, including in some cases that are nonresponsive to anti-VEGF. Despite the improved safety profile of these treatments compared to intraocular injection, they still present a risk for adverse effects, particularly cataract formation and elevated IOP, which must be considered prior to use, particularly in phakic patients.

### 4.4. Kinin-Kallikrein System Inhibitors

Recent studies have also focused on the potential role of the related kinin–kallikrein system (KKS) in retinal inflammation and vasodilation. Originally discovered in 1908, the KKS includes precursors known as high and low molecular weight kininogen (HMWK and LMWK, respectively), which are converted to the kinins by the activity of two separate serine proteases: tissue and plasma kallikrein [101]. The kallikreins are derived when prekallikreins of primarily hepatic origin are activated by factor XIIa. Kallikriens are increased in diabetic rat eyes and are implicated in angiogenesis [102,103,104]. Kallikrein activity on LMWK leads to formation of kinins including kallidin, and HMWK cleavage leads to formation of bradykinin. Alternatively, bradykinin may come from conversion of kallidin. Kallidin and bradykinin can activate bradykinin-2 receptors (B2R), a ubiquitously expressed G-protein coupled receptor that facilitates vasodilation [105]. Reports suggest that B2R may promote beneficial effects [106] but evidence also exists that the B2R receptor facilitates increased retinal vascular permeability [107]. Furthermore, cleavage of an arginine residue by carboxypeptidase activity in response to inflammatory conditions converts the kinins, bradykinin and kallidin, into the bradykinin-1-receptor (B1R) agonists des-Arg^9^-BK and Lys-des-Arg^9^-BK, respectively [108,109]. Unlike B2R, B1R is expressed at very low levels under normal physiological conditions, but is robustly upregulated in response to inflammation, and resists internalization in response to agonist binding, contributing to chronic inflammation [105]. B1R is upregulated in diabetic animal models and in cadaveric tissues of patients with either type 1 or type 2 diabetes [110,111]. Susceptibility to edema is also increased in mice overexpressing B1R, while it is decreased in knockout animals [112,113]. Furthermore, the B1R antagonist attenuates streptozotocin-mediated murine hyperglycemia, R-954 suggesting this therapy could have broader beneficial outcomes on diabetes then retinopathy [104]. Collectively, these studies suggest therapeutic potential for treatments with KKS targets.

KKS components are implicated in human studies of diabetes as well. Plasma prekallikrein was elevated in type I diabetic patients with either non-proliferative or proliferative DR [114], while plasma tissue kallikrein and plasma prekallikrein were both elevated in patients with type II diabetes [115]. Retinal vascular permeability was increased following intravitreal injection of plasma kallikrein or bradykinin, but was rescued by systemic administration of the plasma kallikrein inhibitor, 1-benzyl-1*H*-pyrazole-4-carboxylic acid 4-carbamimidoyl-benzylamide [116]. Additionally, kallikrein activity, factor XIIa production, and vascular permeability are all increased by intravitreal carbonic anhydrase-I injection in rats, and increased intravitreal carbonic anhydrase-I has been observed in patients with DR suggesting a carbonic anhydrase-mediated increase of KKS pathway in DR/DME [117].

KKS is a particularly promising target for therapeutic intervention in that it appears to be mechanistically distinct from VEGF, and may therefore be an effective alternative for the subset of patients who do not respond to anti-VEGF treatments [101]. However, despite their potential, KKS-targeted drugs have only recently garnered significant interest as potential therapies, and application of these drugs to ophthalmic disease remains rare. KalVista Pharmaceuticals initiated a multicenter phase 1 clinical trial in 2014 of a plasma kallikrein inhibitor, KVD001, administered by intravitreal injection, for the treatment of DME. Several novel kallikrein inhibitors are also either in clinical trials or have been approved for non-DR pathologies. DX-88/ecallantide is a kallikrein antibody peptide fragment that is FDA approved to treat hereditary angioedema (HAE), a condition in which bradykinin production is deregulated due to a mutation in the C1 inhibitor gene, leading to potentially fatal edema [118]. Other kallikrein inhibitors under development for the treatment of HAE include the Shire anti-plasma kallikrein monoclonal antibody DX-2930 [119], and the BioCryst small molecule kallikrein inhibitors BCX7353 and BCX4161/avoralstat. Additionally, the B2R antagonist, icatibant (Shire), has been the subject of clinical trials both for HAE, and for ACE inhibitor-induced angioedema, and received FDA approval to treat acute HAE in 2011. While none of the HAE KKS inhibitors other than KDV001 are currently in clinical trials for ophthalmic disease, they raise the exciting possibility of using novel KKS inhibitors to treat DR/DME.

### 4.5. Renin-Angiotensin System Inhibitors

The renin-angiotensin system (RAS) is a major regulator of blood pressure through mediating renal sodium reabsorption and vascular tone, and is a target for DR therapies at multiple points. RAS includes a precursor, angiotensinogen, which is converted to angiotensin I, then angiotensin II by renin and angiotensin converting enzyme (ACE), also known as kininase II [120]. Angiotensin II is a vasoconstrictor and promotes secretion of aldosterone, which increases sodium reabsorption in the kidney. Additionally, a role for RAS in angiogenesis has been identified, leading to both animal studies and clinical trials investigating the therapeutic potential of RAS inhibitors in DR/DME [121]. The angiotensin II type I inhibitor, candesartan, reduced retinal vascular permeability (RVP) in rats treated with angiotensin II, or a streptozotocin induced model of type I diabetes, by >50% [116]. The DIRECT study of >1400 patients found that oral candesartan reduced retinal microaneurysm progression in both type I and II diabetic patients but the therapy was not associated with increased DR regression, reduced DR progression, or decrease in incidence of DME [122]. Surprisingly, a separate multicenter study of 285 patients found that a related angiotensin II inhibitor, losartan, decreased DR progression rates, even when correcting for changes in blood pressure [123]. These studies suggest that angiotensin II inhibitors may be effective against DR in a manner distinct from their anti-hypertensive function.

### 4.6. RAS, KKS, and ACE

Finally, evidence from clinical trials indicates efficacy of specific RAS targets for DR treatment, potentially through crosstalk with KKS. Specific inhibitors to ACE (ACE inhibitors) are among the most commonly prescribed drugs in the USA for their anti-hypertensive effects in preventing conversion of angiotensin I to angiotensin II. However, ACE also acts on KKS components, decreasing B1R expression, and inactivating the kinins [124,125]. While the possibility of ACE inhibitor-induced permeability and/or angioedema due to elevated kinin levels has been raised, ACE inhibitors have nonetheless generated interest and shown promise for DR/DME treatment [126,127,128].

Previous clinical trials have established a link between reduced hypertension and decreased DR progression in patients with type I or II diabetes [129,130]. The United Kingdom Prospective Diabetes Study (UKPDS) studied >1000 patients and found a decrease in DR progression with an ACE inhibitor and a β_1_-adrenergic receptor antagonist, suggesting that the effect may due to mitigated hypertension rather than an ACE inhibitor-specific effect [129]. However, the large, multicenter ACCORD trials found that the use of various anti-hypertensive drugs including ACE inhibitors to normalize blood pressure (systolic <120 mmHg) did not statistically reduce DR progression in patients with type II diabetes [53]. Several studies have identified a reduction in DR progression in normotensive diabetic patients taking an ACE inhibitor, however, even when controlling for any differences in blood pressure, suggesting a possible therapeutic effect apart from hypertensive control [123,130]. This is supported by a meta-analysis of 21 clinical trials with over 13,000 patients combined, revealing no effect of anti-hypertensive drug treatment on DR progression in those with hypertension, but decreased DR progression and increased regression in normotensive patients [131]. By specific drug class, the association with DR progression was lowest with ACE inhibitors, followed by angiotensin-receptor blockers, β-blockers, and finally with calcium channel blockers [131]. Thus, there may yet be a role for anti-hypertensive drugs in slowing DR progression in normotensive patients. Further studies will be necessary to determine the precise mechanisms of action, whether crosstalk with KKS is involved, and demonstrate effectiveness in a multi-center, longitudinal study.

### 4.7. Angiopoietin

The angiopoietins are a family of growth factors involved in vascular maturation and angiogenesis. Angiopoietin 1 (ang1) and 2 (ang2) have significant but opposing effects when binding to their receptor tyrosine kinase, Tie-2. Ang1 binding promotes vessel stabilization and maturation through Tie-2 phosphorylation and downstream signaling [132,133]. Ang1 has anti-inflammatory and pro-barrier effects on vasculature, and intravitreal administration has been shown to block leukocyte adhesion and inflammatory cytokine upregulation in animal models [134,135]. Conversely, ang2 is a competitive, context dependent Tie-2 antagonist that promotes activity of protein tyrosine phosphatase, receptor Type B, also called human protein tyrosine phosphatase beta (HPTPβ), or the mouse ortholog known as vascular endothelial PTP (VEPTP), to dephosphorylate the Tie-2 receptor and prevent downstream signaling, destabilizing the vessels. This signaling is an essential part of angiogenesis but without VEGF signaling may lead to apoptosis [136,137]. Ang2 is elevated in the vitreous of patients with poorly controlled diabetes and in patients with PDR coincident with elevated VEGF compared with either control patients or patients with NPDR [138,139]. When simultaneously present with VEGF, ang2 promotes angiogenesis and vascular sprouting [136,138]. Several drugs currently in pre-clinical development by Akebia Therapeutics specifically target ang2 mediated Tie-2 signaling inhibition, and have the potential to decrease pathogenic angiogenesis and permeability associated with DR/DME. Additionally, an angiopoietin peptibody (peptide fused to an antibody Fc domain), trebananib (Amgen), targets ang1 and 2 and remains in clinical trials for its antiangiogenic effects in various forms of cancer. However, no trebananib trials are currently underway in ophthalmic disease.

### 4.8. Non-Steroidal Anti-Inflammatory Drugs

As previously discussed, accumulating evidence suggests that early stages of DR and DME are driven by inflammation [12,140,141], suggesting that modifiers of either immunity or inflammatory molecules could represent an effective therapeutic strategy. The Early Treatment Diabetic Retinopathy Study (ETDRS) investigated whether patients with NPDR or mild PRD would be protected from progression to more severe PDR and visual impairment by a daily oral regimen of the non-steroidal anti-inflammatory drug (NSAID), aspirin. While the study determined from nearly 4000 patients that at the 650 mg per day dose, aspirin did not prevent or delay disease progression [142], subsequent clinical trials with other NSAIDs have successfully attenuated DR/DME symptoms.

Ketorolac is an NSAID prostaglandin inhibitor widely used for pain relief in a variety of medical procedures including pan-retinal photocoagulation [143,144,145]. It is a member of a specific subclass of NSAIDs (coxibs) that target cyclo-oxygenase (COX) enzymes [146] responsible for prostaglandin production [146]. Ketorolac decreases vitreous inflammatory cytokine concentration and increases visual acuity when given topically, or by intravitreal administration in humans [147,148]. The eye drop form of ketorolac (Acuvail, Allergan) is currently in additional single center clinical trials to gauge intraocular concentrations of the drug and various inflammatory mediators including prostaglandins in patients with DR. Additionally, both ketorolac and the coxib, nepafenic, have been investigated as possible treatments for DME. It should be noted however that the recent DRCR protocol R study failed to show a decrease in retinal volume in patients treated with topical nepafenic [149]. A third coxib, diclofenac, has also been tested pre-operatively in a single center trial for reduction of DME following cataract removal in patients with diabetes. Coxibs and specifically ketorolac have a history of occasional serious side effects [150] but have been well tolerated in ophthalmic applications [151]. They represent a promising new therapy for reducing inflammatory mediators of disease without the risks of cataract formation and elevated IOP associated with corticosteroids [152].

### 4.9. Antibiotics and Immunosuppressants

Similar to NSAIDs, antibiotics have also shown therapeutic promise in reducing DR related inflammation. As part of the early inflammation associated with DR, resident macrophages known as microglia can become activated and release inflammatory cytokines including NF-κB, caspase-3, and IL-1β, leading to leukocyte adhesion, apoptosis, and progressive disease [71,135,153]. Minocycline treatment has been shown to decrease retinal cytokine levels in diabetic rats [153], and an early clinical trial demonstrated decreased retinal thickness and vascular leak, and improved visual acuity following an oral minocycline regimen [154]. A more recent study in diabetic rats suggests that minocycline may act at least in part by attenuating the diabetes-mediated upregulation of poly [ADP-ribose] polymerase 1 (PARP-1), an enzyme involved in DNA repair [155]. PARP-1 is thought to promote apoptosis in response to extensive DNA damage sustained within the diabetic retina. Previously, minocycline has been identified as cytoprotective [156], and may significantly decrease apoptosis [155].

Squalamine is a novel antibiotic named for its source organism, the dogfish shark (*Squalus* acanthias) [157]. In addition to its antibiotic effects, squalamine is an inhibitor of angiogenesis, and has been shown to reduce neovascularization in multiple rodent models of ocular vascular disease [158,159]. A topical form of the drug has also been included in single center human clinical trials for treatment of PDR induced neovascularization in type I or II diabetic patients.

Immunosuppressant drugs have also had success in treating DR/DME. Sirolimus (also known as rapamycin) is an immunosuppressant derived from *Streptomyces hygroscopicus* bacteria, which is used to prevent rejection following organ transplant [160]. In addition, sirolimus and its derivatives are known to have anti-angiogenic and anti-proliferative properties. Proliferation and VEGF expression are decreased in sirolimus-treated cells [161], and oral rapamycin has also been shown to decrease retinal VEGF concentrations in streptozotocin treated rats [162]. Additionally, rapamycin and a related immunosuppressant, everolimus, reduced neovascularization in a mouse oxygen induced retinopathy model [163]. Preliminary clinical trials report increased visual acuity and decreased retinal thickness in type I and II diabetes patients after sirolimus injection every 2 months, or 90 days after a single injection [164]. Sirolimus is perhaps best known as an inhibitor of mammalian target of rapamycin (mTOR), a kinase initiating a signaling cascade necessary for inflammation and that promotes growth in response to energy, processes important in angiogenesis [165]. Future studies will be needed to further elucidate the potential of mTOR inhibitors to treat DR.

### 4.10. Antioxidants

Oxidative stress, defined as a persistent change in the NADH/NAD^+^ ratio, is thought to contribute to DR and the permeability of the retinal vasculature [8]. Changes in oxidative stress can result from various diabetes-related metabolic changes, including mitochondrial metabolism and polyol pathway flux, and can lead to the formation of reactive oxidative species. Recent studies have implicated changes in photoreceptors as a significant source of free radicals and oxidative stress [166]. Antioxidants neutralize reactive oxidative species and may be therapeutically beneficial, yet previous clinical trials have failed to show an association between antioxidants and incidence of DR [167,168]. However, this may be the result of other factors such as insufficient dosing or limited bioavailability of the chosen anti-oxidants. A more recent trial involving patients with type 1 or 2 diabetes and no, mild, or moderate NPDR demonstrated that visual function was preserved after taking an antioxidant cocktail for six months compared to placebo [169]. Further research into the efficacy of antioxidants exploring a variety of treatment paradigms is warranted.

### 4.11. Vitreomacular Adhesion and Vitriol Viscosity Inhibitors

Finally, a novel class of drugs has recently emerged targeting vitreomacular adhesion (VMA). VEGF released as a result of DR can accumulate in the vitreous and encourage neovascularization from the proximal retina to penetrate out into the vitreous. The vitreous acts as a scaffold for these new vessels, and the resulting force exerted on the retina can cause vision-threatening retinal detachment. In a study of 114 non-proliferative DR patients, those with posterior vitreous detachment (PVD) were found to have a drastically lower rate of progression to PDR than patients without PVD, presumably due to lack of proximity between the retina and vitreous, suggesting that intentional induction of PVD could be a therapeutic strategy [170]. The Vitreoretinal Technologies carbomide drug, Vitreosolve, was an early therapeutic attempt given through intravitreal injection, which entered multicenter, phase III clinical trials for the induction of PVD to decrease progression to PDR. While initially promising, the studies were terminated after reporting a non-significant incidence of PVD [171]. However, subsequent drugs in this class have been more successful. Ocriplasmin (ThromboGenics) is a protease delivered by intravitreal injection that has been shown to decrease vitreous viscosity and increase vitreoretinal separation, both in postmortem human and live murine models [172,173]. Efficacy in human patients has been confirmed in multicenter clinical trials involving >600 patients [174,175], and as of 2012, ocriplasmin is FDA approved for the treatment of symptomatic vitreomacular adhesion.

Finally, Luminate (Allegro Ophthalmics) is an anti-integrin peptide currently in multicenter clinical trials for non-proliferative DR and DME. Integrins are transmembrane mediators of cell-extracellular matrix interactions including vitreoretinal adhesion, which also play a role in VEGFR2 activation by VEGF, and angiogenesis [176]. Specific integrin inhibitors have demonstrated effectiveness in preventing proliferation in cells, as well as neovascularization in an OIR rodent model [177,178]. Luminate is distinct from ocriplasmin in that it targets both vitreoretinal adhesion and angiogenesis, potentially increasing its effectiveness. Additionally, administration frequency in current clinical trials is less than once a month, suggesting a possible reduction in burden and increase in compliance in patients, compared to anti-VEGF drugs.

## 5. Summary

**Figure 1:**
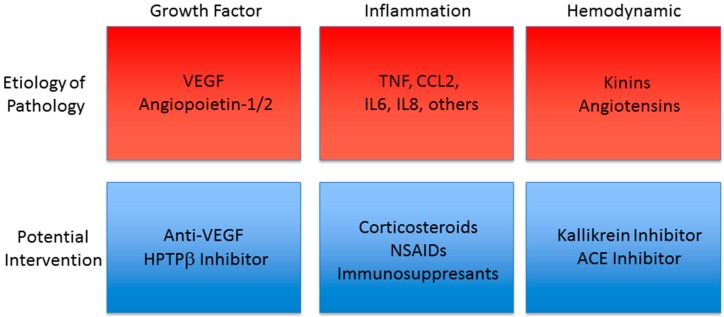
Drug Targets in Trial for Diabetic Retinopathy. Currently, anti-VEGF has shown great effectiveness for many patients with DR. Targeting the Tie-2 receptor signaling through HPTPβ may provide additional benefit. A number of studies demonstrate evidence for inflammation that is targeted through a variety of potential interventions. Hemodynamic control through kallikrein inhibition to prevent kinin production or ACE inhibitors to control angiotensin may also provide potential therapeutic options. The relationship of these drug targets to disease progression is an area of active investigation.

DR and DME are sight-threatening conditions likely to become increasingly common in the coming decades due to the worldwide increase in diabetes incidence. DR and DME manifest as vascular dysfunction through inflammation, pericyte loss and, and breakdown of the BRB and in PDR through neovascularization. Anti-VEGF therapies reveal that targeting this vascular dysfunction can lead to significant improvement in visual function and prevent disease progression (Figure 1). Current treatments including intraocular corticosteroid injections and retinal photocoagulation are associated with significant side effects, and while anti-VEGF treatments have revolutionized DR/DME care, they are invasive, require frequent administration, and are ineffective in a significant subset of patients. Several novel treatments in pre-clinical or clinical trials have the potential to meet currently unmatched needs in DR/DME treatment. These include corticosteroid-releasing implants, which may be particularly useful in pseudophakic patients, and treatments targeting KKS. Stabilizers of vasoprotective Tie-2 signaling have yielded promising results in pre-clinical development, and represent a strategy for preventing disease-associated endothelial dysfunction. Additionally, exciting clinical trial data has been obtained from targeting the inflammation characteristics of early disease with members of multiple drug classes including NSAIDs, immuno-suppressants, and antibiotics. Finally, physical separation of the retina from the vitreous via intentional induction of PVD may provide another potential therapeutic approach. These emerging treatments raise the intriguing and exciting possibility of future DR/DME therapies that are less invasive, more effective, and provide multiple treatment options to reach more individuals with disease.

## Figures and Tables

**Table 1:** Selected pharmacological therapies for the treatment of DR/DME. List of various pharmacological agents indicated for treatment of DR/DME along with drug class, company, clinical trial status, and FDA approval information. Drugs listed as currently in trials for DR/DME must have been listed in a DR/DME specific trial in May 2016 on www.clinicaltrials.gov.

Drug	Class	Company	Currently in Listed Clinical Trials for DR/DME?	FDA Approval for DR or DME?	FDA Approved for Other Condition?
Bevacizumab	Anti-VEGF	Genentech (South San Francisco, CA, USA)	Yes (NCT02462304)	No	Yes (Various forms of cancer)
Ranibizumab	Anti-VEGF	Genentech	Yes (NCT02462304)	Yes	Yes (Neovascular Age-Related Macular Degeneration)
Aflibrocept	Anti-VEGF (VEGF-Trap)	Regeneron (Tarrytown, NY, USA)	Yes	Yes	Yes (Neovascular Age-Related Macular Degeneration)
Pegaptanib	Anti-VEGF	Pfizer (New York, NY, USA)	No	No	Yes (Neovascular Age-Related Macular Degeneration)
PAN-90806	Anti-VEGF	PanOptica (Bernardsville, NJ, USA)	Yes (NCT02475109)	No	No
Infliximab	TNF-α Inhibitor	Janssen Biotech (Horsham, PA, USA)	No	No	Yes (Crohn′s Disease, Ulcerative Colitis, and Various forms of Arthritis)
Adalimumab	TNF-α Inhibitor	AbbVie (North Chicago, IL, USA)	No	No	Yes (Various Autoimmune Disorders Including Arthritis)
Ozurdex	Corticosteroid Implant (dexamethasone)	Allergan (Parsippany-Troy Hills, NJ, USA)	No	Yes	Yes (Uveitis)
Retisert	Corticosteroid Implant (fluocinolone)	Bausch & Lomb (Rochester, NY, USA)	No	No	Yes (Uveitis)
Iluvien	Corticosteroid Implant (fluocinolone)	Alimera Sciences (Alpharetta, GA, USA)	Yes	Yes	No
Candasartin	Angiotensin Receptor Blocker	AstraZeneca (London, UK), Generics	No	No	Yes (Hypertension and Heart Failure)
Losartan	Angiotensin Receptor Blocker	Merck (Kenilworth, UK), Generics	No	No	Yes (Hypertension, Diabetic Nephropathy)
KVD001	Kallikrein Inhibitor	KalVista Pharm. (Porton Down, UK)	No	No	No
DM199	Recomenant human tissue kallikrein-1	DiaMedica (Minneapolis, MN, USA)	No	No	No
Ecallantide/DX-88	Kallikrein Inhibitor	Dyax (Lexington, MA, USA)	No	No	Yes (Herditary Angioedema)
DX-2930	Human monoclonal anti-Kallikrein antibody	Shire (Dublin, Ireland)	No	No	No
BCX7353	Kallikrein Inhibitor	BioCryst (Durham, UK)	No	No	No
Avoralstat/BCX4161	Kallikrein Inhibitor	BioCryst	No	No	No
Icatibant	Bradykinin receptor-2 antagonist	Shire	No	No	Yes (Herditary Angioedema)
Enalapril	ACE Inhibitor	Multiple	No	No	Yes (Hypertension)
Lisinopril	ACE Inhibitor	Multiple	No	No	Yes (Hypertension, Heart Failure, Acute Myocardial Infarction)
AKB-9778	Tie-2 Activator	Akebia Therapeutics (Cambridge, MA, USA)	No	No	No
AKB-9875	Tie-2 Activator	Akebia Therapeutics	No	No	No
AKB-9089	Tie-2 Activator	Akebia Therapeutics	No	No	No
HPTPβ Antibody	Tie-2 Activator	Akebia Therapeutics)	No	No	No
Trebananib	Angiopoietin Blocker	Amgen (Thousand Oaks, CA, USA)	No	No	No
Ketorolac	NSAID (coxib)	Roche (Basel, Switzerland)	Yes (NCT01609881)	No	Yes (Postoperative Ophthalmic Inflammation)
Nepafenic	NSAID (coxib)	Alcon (Hunenberg, Switzerland)	No	No	Yes (Postoperative Ophthalmic Inflammation)
Diclofenac	NSAID	Mutiple	Yes (NCT01694212)	No	Yes (Analgesic, Osteoarthritis)
Minocycline	Antibiotic	Multiple	No	No	Yes (Acne, Various Bacterial Infections)
Squalamine	Anti-microbial	Ohr Pharm. (New York, NY, USA)	Yes	No	No
Rapamycin (Sirolimus)	Immunosuppresant/mTOR Inhibitor	Pfizer	No	No	Yes (Organ Transplant Immunosuppresion, Lymphangioleiomyomatosis)
Everolimus	Immunosuppresant/mTOR Inhibitor	Novartis (Basel, Switzerland)	No	No	Yes (Organ Transplant Immunosuppresion, Various Neuroendocrine tumors)
Vitreosolve	Posterier Vitreous Detachment Agent	Vitreoretinal Tech. (Irvine, CA, USA)	No	No	No
Ocriplasmin	Posterier Vitreous Detachment Agent	ThromboGenics (Leuven, Belgium)	Yes	No	Yes (Symptomatic VMA)
Luminate	Anti-Integrin/Posterier Vitreous Detachment Agent	Allegro Ophthalmics (San Juan Capistrano, CA, USA)	Yes (NCT02435862)	No	No

## Abbreviations

ACE	Angiotensin Converting Enzyme
Ang1	Angiopoietin-1
Ang2	Angiopoietin-2
B1R	Bradykinin-1 Receptor
B2R	Bradykinin-2 Receptor
BRB	Blood Retinal Barrier
COX	Cyclo-oxygenase
Coxib	COX Inhibitor
DME	Diabetic Macular Edema
DR	Diabetic Retinopathy
HAE	Hereditary Angioedema
HMWK	High Molecular Weight Kininogen
HPTPβ	Human Protein Tyrosine Phosphatase β
ICAM-1	Intracellular Adhesion Molecule-1
IOP	Intraocular Pressure
KKS	Kinin-Kallikrein System
LMWK	Low Molecular Weight Kininogen
NF-κB	Nuclear Factor Kappa B
NSAID	Non-Steroidal Anti-Inflammatory Drug
PVD	Posterior Vitreous Detachment
RAS	Renin-Angiotensin System
RPE	Retinal Pigmented Epithelium
TJ	Tight Junction
TNF-α	Tumor Necrosis Factor-Alpha
VEGF	Vascular Endothelial Growth Factor
VMA	Vitreomacular Adhesion
ZO-1, 2	Zonula Occludens-1, 2
